# Can long-acting growth hormone improve adherence in pediatric growth hormone deficiency?

**DOI:** 10.1007/s12020-026-04661-0

**Published:** 2026-05-28

**Authors:** Chiara Rodaro, Gianluca Tamaro, Alice Fachin, Francesca Nicolardi, Silvia Tommasi, Gianluca Tornese

**Affiliations:** 1 Department of Medicine, Surgery and Health Sciences, University of Trieste, Trieste, Italy; 2 Institute for Maternal and Child Health IRCCS Burlo Garofolo, Trieste, Italy

**Keywords:** Growth hormone deficiency, Long-acting growth hormone, Adherence, Compliance, Treatment burden, Pediatric endocrinology

## Abstract

Growth hormone deficiency (GHD) in childhood and adolescence requires long-term therapy with recombinant human growth hormone (rhGH). Although daily injections are highly effective, they impose a considerable treatment burden that often leads to suboptimal adherence and compromised growth outcomes. Reported non-adherence rates vary widely, largely due to heterogeneous definitions and assessment methods. Adherence is influenced by multiple factors, including device usability, psychosocial context, family engagement, and the level of support from health care professionals. Although Long-Acting Growth Hormone (LAGH) preparations, administered once weekly, have demonstrated efficacy and safety comparable to daily rhGH in clinical trials, adherence in these settings may be overestimated. The key question now is whether LAGH can truly enhance treatment adherence in real-world settings. This narrative review summarizes the very limited data currently available on adherence to LAGH in both treatment-naïve patients and those switching from daily rhGH, highlighting methodological heterogeneity and the absence of robust long-term outcomes. Rather than providing definitive conclusions, our aim is to map existing evidence, identify critical gaps, and outline priorities for future real-world and longitudinal studies evaluating adherence, persistence, and clinical consequences of poor adherence in LAGH users.

## Introduction

Daily rhGH has been the standard of care for children with GHD for four decades, leading – when administered consistently – to improved growth velocity, near-target adult height attainment, and favorable long-term metabolic outcomes [1]. However, treatment success is tightly linked to adherence [2], which is frequently suboptimal in real-world practice [3]. In this context, “adherence” denotes the extent to which a patient’s behavior aligns with agreed therapeutic recommendations, whereas “compliance” implies a more passive acceptance of instructions [4]. In pediatric endocrinology, adherence is particularly critical because children depend on caregivers, and family dynamics strongly shape day-to-day treatment implementation [5].

### Adherence issues in daily GH therapy

Maintaining high adherence to daily injections is particularly difficult in the setting of long-term therapy extending over many years and the psychological burden of chronic treatment during childhood and adolescence. Reported non-adherence rates vary widely – from roughly 5% to over 80% [5, 6] – depending on definitions and measurement methods, with “good adherence” usually defined as receiving more than 85% of prescribed injections (i.e., fewer than one missed dose per week) [7]; falling below this threshold is not rare, especially in long‑term treatment extending over several years [8, 9].

### Evidences on poor adherence and impact on growth outcomes 

Crucially, suboptimal adherence is clearly associated with impaired growth outcomes. Children who systematically miss injections show lower annualized height velocity and smaller gains in height SDS compared with those who maintain good adherence, even when treated with similar nominal GH doses [8–10]. Poor adherence also leads to blunted IGF‑1 responses, which may be misinterpreted as GH resistance or underdosing, prompting unnecessary dose escalations or additional diagnostic testing [6]. Beyond growth, poor adherence carries important practical and relational consequences. It is associated with increased healthcare utilization (more frequent visits, repeated investigations, changes of preparation or device), higher costs, and potential erosion of trust in the therapeutic relationship when clinicians and families struggle to explain suboptimal outcomes [5, 8, 11, 12, 13]. These observations illustrate why a basic understanding of adherence patterns and their consequences on height outcomes is essential.

### Methods to assess adherence: strength and limitations 

Nevertheless, assessing adherence in pediatric GH therapy is challenging; it can be assessed using several complementary methods, each with specific strengths and weaknesses. Patient- and caregiver-reported measures (diaries, questionnaires, interviews) are valuable to understand reasons for non-adherence and perceived treatment burden, but they systematically overestimate adherence due to recall and social desirability bias [14, 15]. Objective electronic injection devices provide detailed dosing histories and allow direct linkage between missed doses and auxological or biochemical outcomes [16], but they are not available for all formulations and may not be routinely used in every setting. Pharmacy refill or dispensing data are easy to obtain and suitable for large populations [9, 12, 17], yet they only measure medication possession and cannot distinguish between doses dispensed and doses actually administered. A seminal study by Cutfield et al. highlighted that missed GH injections are common and often go unrecognized in routine care, as poor adherence is rarely disclosed and remains difficult to detect without objective monitoring [12]. More recent surveys show that caregivers report nearly twice the rate of poor adherence compared with physicians’ estimates, suggesting under-recognition by clinicians and/or reluctance among families to disclose missed doses [18]. As a result, reported adherence rates must always be interpreted in the light of the underlying measurement method and the adherence threshold applied.

### Determinants of adherence in pediatric GH therapy 

Multiple, often interacting, factors contribute to suboptimal adherence, including the cumulative burden of daily injections, needle anxiety, device usability issues, socioeconomic and family challenges, declining motivation during adolescence, inadequate training, and misconceptions about the consequences of missed doses [5, 6, 15–19]. A comprehensive biopsychosocial approach – emphasizing early identification of psychosocial barriers such as family stress, treatment fatigue, or inaccurate beliefs about GH therapy – has long been advocated as essential to prevent poor adherence and support long-term treatment success [17]. Real-world data illustrate these challenges. In an Italian survey, nearly one in four children on daily rhGH misses at least one injection per week, most often due to being away from home, forgetfulness, feeling unwell, pain, prescription delays, device malfunction, or lack of refrigerator access [15]. Interventions to improve adherence have focused on education and skills training, progressive patient and caregiver engagement, individualized barrier assessment, multidisciplinary support, and technological aids such as electronic autoinjectors [5, 13, 15], while validated patient-reported outcome instruments (such as the GHD-CIM [Child Impact Measure], CTB [Child Treatment Burden], and GHD-PTB [Parent Treatment Burden]), help quantify the physical, social, and emotional burden of GH therapy on both children and caregivers [20, 21].

### Rationale for long-acting GH formulations 

Beyond these barriers, children, adolescents, and caregivers consistently express a preference for fewer injections. In discrete choice experiments, injection frequency emerges as the dominant driver of treatment preference, with weekly, biweekly, or monthly regimens strongly favored over daily injections [22], particularly among adolescents who are especially vulnerable to treatment fatigue and declining adherence [15]. Notably, 87% of the reasons for non-adherence identified in the Italian survey [15] could, at least in part, be alleviated by switching to a weekly regimen, which also offers a wider dosing window (2–3 days to accommodate delayed injections) and greater flexibility than daily therapy [23]. Within this framework, long-acting growth hormone (LAGH) formulations represent a promising strategy to reduce treatment burden and potentially enhance adherence [2, 24, 25]. Patients at highest risk of poor adherence – and therefore prime candidates for LAGH – include those with multiple comorbidities requiring multiple medications (e.g., panhypopituitarism), needle phobia or behavioral disorders, very young children, adolescents, children from separated families, those with frequent travel routines, and those whose activities regularly interfere with fixed injection routines [23, 25, 26]. In daily practice, a structured screening tool (such as the checklist presented in Table 1) may help clinicians systematically identify these high‑risk profiles and support shared decision‑making when considering LAGH therapy.

**Table 1:** Screening form for LAGH (long-acting growth hormone) eligibility

Are the patient or the cargivers afraid of needles or injections?	
Does the patient have neurodiversity or behavioral disorders (e.g., ASD, ADHD) that may increase distress with injections?	
Does the patient take part in scout or similar group activities (e.g., camps, overnight trips, frequent sleepover)?	
Does the patient have more than one home (e.g., separated parents)?	
Does the family travel frequently (for work, holidays, sports, etc.)?	
Are there relevant socioeconomic difficulties that may increase treatment burden?	
Is the child on multiple daily medications/a complex regimen (e.g. panhypopituitarism)?	
Are there possible or known adherence issues?	
Is the patient an adolescent?	
Is the patient very young (but > 3 years old)?	

### Available LAGH preparations and scope of this review 

Currently available LAGH molecules widely used in international practice [23–27] include lonapegsomatropin [28], somapacitan [29], and somatrogon [30]. Other agents, such as PolyEthylene Glycol(PEG)–Conjugated Recombinant Human Growth Hormone (PEG-rhGH), are available only in selected markets [31]. Randomized controlled trials have demonstrated the non-inferiority of LAGH compared with daily rhGH in annualized height velocity, height standard deviation score (SDS), and Insulin-like Growth Factor 1 (IGF-1) responses, with comparable safety profiles [23–25]. However, despite a growing body of data on the efficacy and safety of LAGH, adherence has rarely been a primary endpoint, and there are almost no long-term real-world data comparing adherence to daily rhGH versus LAGH or describing the clinical and biochemical impact of poor adherence under weekly regimens. Existing information is fragmented across randomized trials, observational cohorts, preference studies, and economic models, each using different and often indirect measures of adherence. The objective of this narrative review is therefore not to provide a general, exhaustive overview of adherence to GH therapy in pediatric GHD, but to systematically collate and critically appraise the limited evidence available on adherence with LAGH, including comparisons with daily formulations where possible. Building on the concepts outlined above, the review aims to map current data on adherence in LAGH users, explore how weekly regimens and specific device attributes may help overcome known barriers, and propose a conceptual framework and research agenda for future real-world and longitudinal studies.

We conducted a structured narrative review of the literature in PubMed, Embase, and Scopus, covering publications up to January 2026. Search terms included “growth hormone deficiency”, “adherence”, “compliance”, “long-acting growth hormone”, “somapacitan”, “somatrogon”, “lonapegsomatropin”, “PEG-rhGH”. Randomized controlled trials and observational studies were included. The reference lists of selected articles were also screened to identify additional relevant studies. In total, we identified 30 primary studies reporting data on adherence or adherence-related outcomes with LAGH in pediatric populations, which are summarized in Table 2 [2, 3, 13, 32–58].

**Table 2:** Summary of published studies reporting adherence and adherence-related outcomes with long-acting growth hormone in pediatric growth disorders

Author / Journal / Year	Molecule	Sample Size	Study Design	Endpoints	Main Findings on Adherence and Adherence-Related Outcomes
Akhtar S et al. Med Devices 2024 [3]	Somapacitan vs. somatrogon (device comparison)	70 participants: 33 adolescents with growth-related disorders and 37 caregivers	Randomized crossover simulated-use study	Device preference, ease of use, injection performance	Somapacitan device was preferred over somatrogon (84.3% vs. 12.9%, *p* < 0.0001) and rated easier to use (98.6% vs. 74.3%) by patients and caregivers. These findings support a potential adherence-related advantage but do not directly measure adherence.
Al-Agha AE et al. Saudi Med J 2025 [32]	Somapacitan (naïve and switch)	184 children with GHD	Real-world observational study, 72 weeks	HV, IGF-1, safety, adherence	Overall adherence was 99.58% (treatment-naive 99.43%; switch 99.92%).
Cai W et al. Biomark Med 2025 [33]	PEG-rhGH (low- and high-dose) vs. daily rhGH	192 children with short stature	RCT, 6 months	Growth velocity, bone markers, metabolic and thyroid markers, adverse events, adherence	Treatment adherence was significantly higher in both weekly long-acting GH groups than in the daily rhGH group (100% vs. 90.62%, *p* = 0.028).
Chang W, et al. Front Pediatr. 2025 [13]	LAGH (not specified) vs. daily rhGH	8,621 children with short stature (1,856 LAGH; 6,765 daily rhGH)	Real-world retrospective observational study	Adherence rate, factors associated with adherence	Mean adherence was high overall and significantly higher with LAGH than with daily rhGH (94% vs. 91%, *p* < 0.001); more patients in the LAGH group achieved adherence thresholds of ≥ 90%, ≥ 85%, and ≥ 80%.
Chen JV et al. J Endocr Soc 2025 [34]	Somapacitan, lonapegsomatropin, and somatrogon (device profiles)	100 participants: 50 adults, 25 caregivers, and 25 caregiver/adolescent dyads	Cross-sectional discrete choice experiment	Device preference, predicted choice probability	Somapacitan device profile had the highest predicted choice probability (82% vs. 16%), mainly driven by lower pain, fewer injections per dose, and shorter preparation time. These findings support a potential adherence-related advantage but do not directly measure adherence.
Cohen-Sela E et al. Endocr Pract 2025 [35]	Somatrogon	65 pediatric endocrinologists reporting on at least 874 patients	Cross-sectional national survey	Prescribing patterns, satisfaction, perceived adherence, adverse events	Physicians generally reported good perceived adherence with somatrogon, with 54.8% stating that patients rarely missed a dose and 33.9% stating that patients had not missed any doses; however, 59.7% reported at least one case of reversion to daily rhGH.
Deal CL et al. J Clin Endocrinol Metab 2022 [36]	Somatrogon vs. daily rhGH	224 children with GHD (109 somatrogon; 115 daily rhGH)	Phase 3 non-inferiority RCT, 52 weeks	Annualized HV, height SDS, IGF-1, safety, adherence	Adherence was very high in both groups, with mean adherence of 99.4% for somatrogon and 99.7% for daily rhGH. The lowest adherence rate observed for an individual patient was 87.5% in the somatrogon group and 91.5% in the daily rhGH group.
Fu J, et al. Horm Res Paediatr. 2025 [37]	Somapacitan vs. daily rhGH	103 children with GHD (71 somapacitan; 32 daily rhGH)	Phase 3 RCT (REAL6), 52 weeks	HV, height SDS, IGF-1 SDS, bone age, treatment burden, safety	Adherence was high in both groups (mean 95.3% with somapacitan and 94.0% with daily rhGH), while treatment burden was significantly lower with somapacitan (GHD-PTB interference and total scores *p* < 0.05).
Gomez R et al. Front Endocrinol 2023 [2]	Somatrogon vs. daily rhGH	24 pediatric endocrinologists from the phase 3 study	Cross-sectional survey	Physician-reported treatment burden, acceptance, perceived adherence	Among participating physicians, 62.5% reported that monitoring adherence required greater or much greater effort with daily rhGH injections than with weekly somatrogon, while 20.8% reported no difference; 33% preferred somatrogon because they believed it would likely improve adherence, and 79.2% considered somatrogon less burdensome overall than daily rhGH.
Jie L et al. J Pediatr Endocrinol Metab. 2026 [38]	PEG-rhGH vs. daily rhGH vs. sequential PEG-rhGH → daily rhGH	105 children with ISS (53 daily rhGH only, 30 PEG-rhGH, 22 sequential)	Single-center retrospective cohort, 48 weeks	Height SDS, HV, IGF-1, bone age, safety, adherence, cost-effectiveness	Median missed injection rates were lowest with PEG-rhGH (0%), intermediate with sequential (0.14%), and highest with daily rhGH (0.27%), and missed injections were the only independent predictor of increase in height SDS in multivariable analysis.
Juul A et al. Eur J Endocrinol. 2026 [39]	Somapacitan vs. daily rhGH with switch extension	62 children born SGA with short stature; 60 entered extension; 55 completed 208 weeks	Phase 2 randomized, open-label, controlled study, 4-year extension	Long-term efficacy and safety, adherence, patient preference	Mean adherence with somapacitan remained high over long-term follow-up (95.3% over 208 weeks). Prior to switching, adherence to daily rhGH ranged from 84.8% to 92.3%, and remained relatively high after switching to weekly somapacitan (80.2–86.0%). PRO showed a strong preference for weekly therapy (87%), with 80% of those preferring somapacitan expecting improved adherence compared to daily rhGH.
Lebl J et al. Cesko-Slovenska Pediatrie. 2024 [40]	Somatrogon (naïve and switch)	166 children and adolescents with GHD (60 treatment-naive; 106 switched)	Real-world retrospective observational study, 1 year	Growth outcomes, adherence, tolerability	About 7% of patients (4/166) reverted from somatrogon to daily rhGH: 2 treatment-naive children because families reported administration difficulties attributed to the formulation, and 2 previously switched patients because of side effects that resolved after returning to daily rhGH.
Luo X et al. Eur J Endocrinol 2017 [41]	PEG-rhGH vs. daily rhGH	Phase 2: 97 children with GHD; Phase 3: 343 children with GHD	Phase 2 and Phase 3 RCTs, 25 weeks	HV, growth parameters, safety, compliance	Compliance was high and broadly comparable between weekly PEG-rhGH (96.9%) and daily rhGH (99.1%).
Maniatis AK, et al. J Endocr Soc. 2022 [42]	Somatrogon vs. daily rhGH	87 children with GHD	Phase 3 two-period crossover RCT, 12 weeks	Life interference, caregiver burden, injection schedule preference, convenience, satisfaction, safety	Somatrogon was associated with a significantly lower treatment burden than daily rhGH (LI total score 8.63 vs. 24.13, *p* < 0.0001). Although fewer than 50% of patients/caregivers preferred the somatrogon pen for some handling tasks, a higher proportion reported greater intention to comply with the somatrogon treatment schedule than with daily rhGH.
Maniatis AK, et al. Horm Res Paediatr. 2022 [43]	Lonapegsomatropin vs. daily rhGH	146 children with GHD (143 switch)	Phase 3 multicenter open-label trial (fliGHt), 26 weeks	Safety, tolerability, HV, height SDS, IGF-1 SDS, treatment preference, treatment burden	Treatment adherence with lonapegsomatropin was very high (mean 98.4%). Most children and parents preferred lonapegsomatropin to previous daily rhGH, and treatment burden significantly decreased after switching (*p* < 0.05).
Maniatis AK, et al. J Clin Endocrinol Metab 2022 [44]	Lonapegsomatropin	298 children and adolescents with GHD from the heiGHt and fliGHt trials	Phase 3 open-label extension trial (enliGHten), 2 years	Long-term safety, efficacy, IGF-1 SDS, convenience, tolerability, compliance	Mean compliance was 98.8%. Switching from daily rhGH or from lonapegsomatropin administered via vial/syringe to the autoinjector significantly improved convenience and overall treatment satisfaction, although results were not stratified by prior treatment modality.
Medina J et al. Patient Prefer Adherence 2025 [45]	Somapacitan vs. lonapegsomatropin (device comparison)	70 participants: 35 adolescents and 35 caregivers	Simulated-use preference study	Usability, device preference, preparation and injection time	Most participants (78.6%) preferred the somapacitan device over the lonapegsomatropin device. Most rated the somapacitan pen as easy to use (97.1%) and easy to learn (95.7%). These findings support a potential adherence-related advantage but do not directly measure adherence.
Miller BS et al. J Clin Endocrinol Metab 2022 [46]	Somapacitan vs. daily rhGH	200 children with GHD (132 somapacitan; 68 daily rhGH)	Phase 3 RCT (REAL4), 52 weeks	HV, height SDS, IGF-1, treatment burden, safety, adherence	Adherence was high in both groups (mean 95.8% for somapacitan vs. 88.3% for daily rhGH), and weekly somapacitan was associated with lower treatment burden, including fewer schedule disruptions and less worry about remembering injections.
Miller BS et al. J Clin Endocrinol Metab 2023 [47]	Somapacitan vs. daily rhGH with switch extension	200 children with GHD (132 somapacitan, 68 daily rhGH); 194 completed 2 years	Phase 3 extension study (REAL4), 2 years	HV, height SDS, IGF-1, treatment burden, safety	Adherence during year 2 was high in both groups (mean 90.3% in the continuous somapacitan group and 88.8% in the switch group). Among caregivers of children who switched from daily rhGH, 90% preferred once-weekly somapacitan and 77.8% of those preferring somapacitan stated that they would be more adherent to the weekly regimen.
Miller BS et al. Eur J Endocrinol. 2025 [48]	Somapacitan vs. daily rhGH with switch extension	200 children with GHD (132 somapacitan, 68 daily rhGH); 188 completed 3 years	Phase 3 extension study (REAL4), 3 years	HV, height SDS, IGF-1, safety	Adherence during year 3 was high in both groups (mean 89.0% in the continuous somapacitan group and 86.0% in the switch group).
Mori J et al., Clin Endocrinol. 2024 [49]	Somapacitan vs. daily rhGH with switch extension	30 children with GHD	Phase 3 RCT sub-analysis (Japanese sub-cohort from REAL4), 104 weeks	HV, height SDS, IGF-1, safety	Adherence was high in both groups (at week 52: mean 98.2% with somapacitan vs. 94.8% with daily rhGH; at week 104: 93.5% for continuous somapacitan and 83.2% in the switch group). In the switch group, 90.9% of caregivers preferred once-weekly somapacitan to daily rhGH, and 60% of those preferring somapacitan stated they would be more adherent to weekly dosing, mainly due to fewer injections and less worry about remembering injections
Sävendahl L et al. J Clin Endocrinol Metab 2020 [50]	Somapacitan vs. daily rhGH	59 children with GHD (45 somapacitan; 14 daily rhGH)	Randomized phase 2 trial (REAL3), 52 weeks	HV, height SDS, HV SDS, IGF-1 SDS, safety, adherence, GHD-CIM	Adherence was high in all groups, with mean diary-recorded adherence of 97.5%, 98.6%, and 96.3% in the somapacitan arms and 91.8% with daily rhGH. Findings support a potential adherence advantage mainly through reduced injection burden and observer-reported benefits rather than through a statistically proven adherence superiority.
Sävendahl L et al. J Clin Endocrinol Metab 2022 [51]	Somapacitan vs. daily rhGH	59 children with GHD (45 somapacitan; 14 daily rhGH); 53 completed 3 years	Randomized phase 2 trial (REAL3), 3-year analysis	HV, height SDS, HV SDS, IGF-1 SDS, IGFBP-3 SDS, GHD-CIM, safety, adherence	Mean adherence remained high and was similar between groups over 3 years (92.2% for pooled somapacitan and 87.2% for daily rhGH). The study suggests a potential adherence-related benefit through lower treatment burden and fewer injections but does not demonstrate statistically significant superiority in adherence.
Sävendahl L et al. J Clin Endocrinol Metab 2023 [52]	Somapacitan vs. daily rhGH with switch extension	59 children with GHD (45 somapacitan; 14 daily rhGH); 50 completed 4 years	Long-term safety extension of the phase 2 REAL3 trial	HV, change in HV SDS, change in height SDS, disease burden, treatment burden, safety, GH-PPQ	Observer-reported outcomes indicated reduced treatment burden after switching from daily rhGH to somapacitan. Most caregivers (81.8%) strongly or very strongly preferred somapacitan, and 88.9% reported that they would be more adherent to somapacitan than to daily rhGH.
Sävendahl L et al. J Endocr Soc 2026 [53]	Somapacitan vs. daily rhGH with switch extension	Children with GHD - Cohort I: 59 originally randomized, 43 completed the 7-year extension; additional safety cohorts enrolled after week 156	Final 7-year analysis of the phase 2 REAL3 trial with long-term safety extension	Long-term efficacy, safety, IGF-1/IGFBP-3, treatment burden, near-adult-height outcomes	Mean adherence to treatment was 87.2% for daily rhGH between weeks 0 and 156; 83.7% at week 156 when they switched to somapacitan; and 85.1% for the pooled somapacitan group.
Tamaro G et al. Front Endocrinol 2026 [54]	Somatrogon (naïve and switch)	40 children/adolescents with GHD (20 treatment-naïve, 20 switched from daily rhGH)	Single-center retrospective real-world observational study	Auxological response, IGF-1, dose adjustments, safety, practical treatment experience	Somatrogon was chosen in 25% of switch patients for poor adherence, with a 6-month height SDS gain (+ 0.17) similar to naive patients (+ 0.19). No patient discontinued or reverted to daily therapy, and about 30% of switch families spontaneously reported better adherence and less injection-related distress.
Thornton PS et al. J Clin Endocrinol Metab 2021 [55]	Lonapegsomatropin vs. daily rhGH	161 treatment-naive prepubertal children with GHD (105 weekly lonapegsomatropin, 56 daily somatropin)	Phase 3 RCT (heiGHt), 52 weeks	Annualized HV, height SDS, IGF-1/IGF-1 SDS, IGFBP-3, bone age/chronological age ratio, safety, immunogenicity, adherence	Overall treatment adherence was very high and similar in both arms (mean 99.6% with lonapegsomatropin and 98.6% with daily somatropin).
Xie L et al., Eur J Pediatr 2024 [56]	PEG-rhGH vs. daily rhGH	95 prepubertal children with ISS (47 PEG-rhGH, 48 daily rhGH)	Two-year real-world retrospective cohort study	Catch-up growth, HV, change in height SDS, BMI SDS, IGF-1 SDS, bone age progression, safety, and self-reported adherence	PEG-rhGH showed significantly fewer self-reported missed doses than daily rhGH in both years (year 1: 0.48 ± 0.82 vs. 11.50 ± 7.83; year 2: 0.30 ± 0.61 vs. 20.24 ± 14.84), corresponding to less than 1% versus 4.4% missed doses overall.
Wu W et al. Adv Ther. 2025 [57]	PEG-rhGH vs. daily rhGH	41,942 children with short stature (GHD, ISS, SGA) from two national databases	Multicenter, retrospective, observational real-world study using registry data (CGSD and CGLS)	Treatment patterns, initial dosing, treatment duration, discontinuation rates, and reasons for discontinuation	Discontinuation was 37.8% in those initiated on PEG-rhGH vs. 81.7% in those initiated on daily GH. The most common reasons for discontinuation were irregular administration (18.3%), limited access to medication (16.9%), and refusal by the child or family (11.2%).
Zadik Z et al. J Pediatr Endocrinol Metab 2023 [58]	Somatrogon	53 children with GHD, 48 entered the 5-year open-label extension	Phase 2 RCT dose-finding trial with long-term open-label extension	HV, height SDS, bone age, IGF-1 SDS, safety, and adherence	Mean adherence of 98% throughout the 5 years of the open-label extension period.

BMI, Body Mass Index; GHD, Growth Hormone Deficiency; GHD-PTB, Growth Hormone Deficiency-Parent Treatment Burden; HV, HV; IGF-1, Insulin-like Growth Factor 1; IGFBP‑3, Insulin‑Like Growth Factor Binding Protein 3; ISS, Idiopathic Short Stature; PEG-rhGH, Polyethylene Glycol–Conjugated Recombinant Human Growth Hormone; rhGH, recombinant human Growth Hormone; LAGH, Long-Acting Growth Hormone; PRO, Patient-Reported Outcome; RCT, Randomized Controlled Trial; SDS, Standard Deviation Score; SGA, Small for Gestational Age

## Results

### RCT in treatment-naïve children

Among treatment-naïve children, phase 3 RCTs consistently show that weekly LAGH achieves high adherence and reduces treatment burden compared with daily rhGH, although differences in adherence are often modest in the trial setting. A review and a meta-analysis found that patients receiving somapacitan had significantly higher odds of being adherent than those on daily rhGH [59, 60]. In the phase 2 REAL3 trial, adherence was numerically higher in those receiving somapacitan (96.3–98.6%) compared with those receiving daily rhGH (91.8%), and in the phase 3 REAL4 trial, mean adherence was 95.8% with weekly somapacitan vs. 88.3% with daily rhGH; weekly treatment was associated with fewer schedule disruptions and less worry about remembering injections, as reflected by lower treatment-burden scores [46, 50]. In the Chinese REAL6 trial, adherence was similarly high with somapacitan and daily rhGH (95.3 vs. 94.0%), but somapacitan significantly reduced parent-reported treatment burden [37]. Similarly, in the Japanese sub-cohort from REAL4, adherence was high in both groups (98.2% weekly, 94.8% daily) [49]. For lonapegsompatropin and somatrogon, treatment-naïve children showed near-perfect adherence in both arms (99.6% weekly vs. 98.6% daily for lonapegsomatropin, 99.4% weekly vs. 99.7% daily for somatrogon), with no clinically relevant difference [36, 55]. Likewise, phase 2 and 3 trials of PEG-rhGH vs. daily rhGH in GHD reported high and broadly comparable compliance (around 97 vs. 99%) [41], and in a 6-month RCT in short stature, PEG-rhGH weekly regimens achieved 100% adherence compared with 90.6% with daily rhGH [33].

Treatment-burden–focused RCTs reinforce these findings. In the crossover life interference study, weekly somatrogon was associated with substantially lower treatment burden than daily rhGH in both children and caregivers, with large differences in life-interference scores despite the short 12-week duration [42]. Overall, these trials indicate that weekly LAGH regimens are at least as acceptable as daily rhGH and often reduce treatment burden, but the magnitude of adherence gains in naïve patients is limited by “ceiling effects” in adherence.

### RCTs and open-label studies of switching from daily rhGH to LAGH

Switching from daily rhGH to LAGH is a common clinical strategy when adherence, burden, or needle-related distress are problematic [61]. In the fliGHt trial, children switching from daily rhGH to weekly lonapegsomatropin showed very high adherence (mean 98.4%), marked reductions in reported treatment burden, and a strong preference for the weekly regimen, with most children and parents wishing to continue therapy beyond the study [43]. Similarly, in REAL4, children who switched from daily rhGH to somapacitan after one year maintained high adherence (around 86–89% in the years following the switch) and showed strong caregiver preference for the weekly regimen; most caregivers who preferred somapacitan stated that they would be more adherent with the weekly schedule, largely due to fewer injections and less worry about missed doses [47–49]. In the REAL3 study, children originally randomized to daily rhGH and then switched to weekly somapacitan showed a reduction in treatment-burden scores, with most parents or caregivers strongly preferring once-weekly injections and indicating that they would be more adherent than with daily rhGH [51–53].

From the physician perspective, a survey among investigators in the phase 3 somatrogon program found that 62.5% perceived that monitoring adherence required greater or much greater effort with daily rhGH injections than with weekly somatrogon, and one third preferred somatrogon because they believed it would likely improve adherence; nearly 80% considered somatrogon less burdensome overall than daily rhGH [2]. Overall, across different molecules, switch studies suggest that changing from daily rhGH to LAGH tends to preserve or improve growth trajectories, while both adherence metrics and PROs consistently favor the weekly regimen, although pre- vs. post-switch adherence is rarely quantified with the same methodology.

### Long-term extension trials with LAGH

Long-term extension trials provide information on the sustainability of weekly regimens. In enliGHten, mean compliance with lonapegsomatropin remained around 99% over two years, including in children previously treated with daily rhGH, with improved convenience and overall satisfaction [44]. In the REAL4 extensions, adherence during years 2 and 3 remained high with somapacitan (around 86–90%) in both continuous and switch groups, and caregiver preference for weekly treatment persisted [47–48]. In the REAL3 study, 3-, 4- and 7-year data show high adherence and stable or reduced treatment burden over time with weekly somapacitan [51–53]. Similarly, in REAL5, once‑weekly somapacitan maintained high adherence over 4 years in children born SGA (mean 95.3% over 208 weeks), including after switching from daily GH, with 87% of respondents preferring weekly injections and 80% expecting better adherence with somapacitan [39]. Long-term data with weekly somatrogon over five years likewise show sustained improvement in height SDS and mean compliance around 98% in children with GHD [58].

### Heterogeneity and limitations of adherence data in RCTs

Across these trials, adherence differences between LAGH and daily rhGH are not uniform. In some naïve-patient RCTs for lonapegsomatropin, PEG-rhGH, and somatrogon, adherence was essentially identical and extremely high in both arms, with mean rates around 97–99% and individual values rarely falling below 85–90% [36, 41, 55]. In others, such as those with somapacitan and the PEG-rhGH study in short stature, weekly regimens showed numerically or statistically higher adherence and fewer missed doses than daily rhGH [33, 46–48, 56].

Similar patterns are seen in switch and extension studies, where adherence with LAGH often approaches 98–99%, but comparisons with daily rhGH are indirect or absent. It is therefore difficult to determine whether observed differences are driven by dosing interval, molecule, device characteristics, or trial-specific factors, and in all cases adherence remains unusually high in both arms compared with real-world practice. Importantly, systematic reviews of pediatric rhGH therapy indicate that daily regimens in prospective studies and RCT-like settings also achieve very high adherence, often above 90–95% at 12 months, particularly when monitored with electronic devices or intensive follow-up [62]. This shared “ceiling effect” for both daily and weekly GH helps explain why between-arm differences in adherence are often small or non-significant in RCTs. Moreover, none of these trials was primarily designed to evaluate adherence: adherence was generally assessed using diaries, device logs, or drug accountability rather than validated adherence instruments, and study populations were highly selected and intensively monitored (e.g. frequent visits, structured education). As a consequence, RCT data are likely to overestimate achievable adherence in routine practice and may underestimate the true real-world advantage of weekly LAGH over daily rhGH.

### Real-world experience with LAGH and adherence-related outcomes

Real-world studies provide complementary information, capturing persistence, switching patterns, and patient-reported experience under less controlled conditions. A large Chinese retrospective cohort of 8,621 children with short stature reported higher mean adherence with LAGH (not specified) compared with daily rhGH (94 vs. 91%) and a greater proportion of patients achieving adherence thresholds ≥ 90%, ≥ 85%, and ≥ 80%; patients on LAGH were 57% less likely to be non-adherent than those on daily therapy, independent of demographic or clinical factors [13]. Consistent with this, a nationwide Chinese registry including 41,942 children reported markedly lower discontinuation in those initiated on PEG-rhGH than in those started on daily rhGH (37.8% vs. 81.7%), with irregular administration, limited access to medication, and child or family refusal as the most frequent reasons for stopping therapy [57]. In another two-year real-world retrospective cohort study, PEG-rhGH showed significantly fewer self-reported missed doses than daily rhGH in both years, corresponding to less than 1% versus 4.4% missed doses overall [56]. In a single‑centre retrospective cohort of 105 children with ISS, median missed injection rates were lowest with weekly PEG‑rhGH (0%), intermediate with sequential PEG‑rhGH to daily rhGH (0.14%), and highest with daily rhGH alone (0.27%); missed injections were the only independent predictor of the increase in height SDS in multivariable analysis, underscoring the central role of adherence for height response [38]. For somapacitan, observational data from routine practice in Saudi Arabia showed very high adherence across both treatment-naïve and switch patients, with mean adherence rates above 95% and low rates of missed doses, though without formal comparison to daily rhGH in the same setting [32].

By contrast, several other real-world series focus primarily on auxology, safety, or prescribing patterns and either do not collect adherence systematically or report only qualitative impressions. Registry data from INSIGHTS-GHT, for example, provide an early picture of real-world LAGH use across pediatric and adult GHD in Germany, describing patient selection, dosing, and early safety, but adherence outcomes were not yet systematically reported [27]. An Italian single-center study of 40 children using somatrogon reported that height SDS gains at 6 months were comparable between switch and treatment-naïve patients; although causality cannot be established, these data are consistent with improved treatment consistency after switching to weekly therapy [54]. In the same study, weekly therapy was chosen in 25% of switch cases specifically because of poor adherence, approximately 30% of switch families spontaneously reported improved adherence and reduced injection-related distress, and none of the patients discontinued or reverted to daily therapy during follow-up [54]. In a Czech real-world cohort of 166 children treated with somatrogon, about 7% reverted to daily rhGH, mainly due to administration issues or adverse events, despite overall favorable growth and tolerability [40]. In a national survey of somatrogon use, most pediatric endocrinologists perceived adherence as good or very good, although nearly 60% had encountered at least one reversion from weekly to daily therapy, highlighting that LAGH is not universally suitable or sufficient to prevent non-adherence [35].

### Device characteristics, preference, and adherence-related outcomes

Beyond dosing frequency, device characteristics appear to play an important role in patient and caregiver preference and may indirectly influence adherence. Observational data in daily rhGH therapy indicate that allowing families to choose their preferred device can reduce non-adherence and improve growth outcomes over three years, supporting the integration of shared decision-making into GH prescribing [8]. In a study of children with GHD, the opportunity to choose the preferred device was associated with markedly lower non-adherence over 3 years and with better growth outcomes [63]. This finding is consistent with evidence that shared decision-making can support engagement and adherence in pediatric GH therapy [6, 64].

In an RCT device-comparison study, adolescents and caregivers preferred the somapacitan device over the somatrogon device in simulated use, rating it easier to use and more intuitive [3]. A similar simulated-use study showed that the somapacitan pen required less training and shorter preparation and injection times than the lonapegsomatropin device, with most participants rating somapacitan as easier to learn and to handle [45]. These experimental findings are consistent with a discrete choice experiment in 100 US patients and caregivers, in which the somapacitan device profile had the highest predicted choice probability (82%) compared with lonapegsomatropin (16%) and somatrogon (2%), driven mainly by lower post-injection pain, fewer injections per dose, and shorter preparation time [34]. Although these studies do not measure adherence directly, they highlight how usability, injection comfort, and preparation time—rather than device electronics or storage conditions—dominate treatment choice and are likely to affect long-term engagement.

As illustrated in Table 3, different LAGH preparations also differ in the weekly injection volume and, in some weight ranges, in the need for one versus two injections [28–30], aspects that should be discussed with families alongside pharmacological properties when selecting a specific LAGH.

**Table 3:** Weekly doses, pen strengths, and injection volumes for once-weekly GH preparations (somatrogon, somapacitan, lonapegsomatropin) at different body weights, calculated using approved weight-based dosing regimens and currently available device presentations

Weight	Somatrogon (0.66 mg/kg/week)			Somapacitan (0.16 mg/kg/week)			Lonapegsomatropin (0.24 mg/kg/week)		
	Somatrogon dose per week	Pen	Volume per week	Somapacitan dose per week	Pen	Volume per week	Lonapegsomatropin dose per week	Pen	Volume per week
**10 kg**	6.6 mg	Ngenla 24 mg [20 mg/1 mL] (up to 12 mg per injection)	0.33 mL	1.6 mg	Sogroya 5 mg [5 mg/1.5 mL] (up to 2 mg per injection)	0.48 mL	2.4 mg	Not available (available ≥ 11.5 kg)	-
**15 kg**	9.9 mg	Ngenla 24 mg [20 mg/1 mL] (up to 12 mg per injection)	0.495 mL	2.4 mg	Sogroya 10 mg [10 mg/1.5 mL] (up to 4 mg per injection)	0.36 mL	3.6 mg	Skytrofa 3.6 mg [11 mg/mL]	0.327 mL
**30 kg**	19.8 mg	Ngenla 60 mg [50 mg/1 mL] (up to 30 mg per injection)	0.396 mL	4.8 mg	Sogroya 15 mg [15 mg/1.5 mL] (up to 8 mg per injection)	0.48 mL	7.6 mg	Skytrofa 7.6 mg [22 mg/mL]	0.345 mL
**50 kg**	33 mg	Ngenla 60 mg [50 mg/1 mL] (up to 30 mg per injection)	0.66 mL (2 injections)	8 mg	Sogroya 15 mg [15 mg/1.5 mL] (up to 8 mg per injection)	0.8 mL	12 mg	Skytrofa 11 mg [22 mg/mL]	0.50 mL
**75 kg**	49.5 mg	Ngenla 60 mg [50 mg/1 mL] (up to 30 mg per injection)	0.99 mL (2 injections)	12 mg	Sogroya 15 mg [15 mg/1.5 mL] (up to 8 mg per injection)	1.2 mL (2 injections)	18 mg	Skytrofa 9.1 mg [22 mg/mL]	0.87 mL (2 injections)

Text in square brackets indicates the concentration of the medicinal product, expressed as total drug content per milliliter of solution. Text in round brackets provides additional practical information about use of the device, such as the maximum amount deliverable per single injection or whether two injections are required to administer the full weekly dose. Doses and volumes are indicative and must always be confirmed and individualized according to the official product information and clinical judgement

#### Transition to adult care and regulatory constraints

 A further consideration is that a substantial proportion of children diagnosed with GHD—ranging from 20% to 87%, depending on etiology and diagnostic criteria—will continue to have persistent deficiency into adulthood [65]. Some LAGH formulations are approved for both pediatric and adult use [66, 67], whereas others are restricted to pediatric indications only. As a result, a paradoxical situation may arise in the transition phase: adolescents stable on weekly LAGH may be forced to switch back to daily rhGH solely due to regulatory constraints. This transition phase is particularly vulnerable, characterized by high rates of therapy interruption, limited follow-up, and poor persistence, with up to 48% of patients experiencing prolonged treatment gaps or “drug holidays” [68–71].

#### Unresolved questions and knowledge gaps

Overall, the available literature does not yet allow a robust quantification of adherence to LAGH or a clear comparison with daily rhGH, nor does it describe the clinical or biochemical sequelae of suboptimal adherence to weekly preparations. This paucity of data is, in itself, a key finding of the present review. Therefore, despite advances in LAGH therapy, several important gaps remain.

1. Standardized and validated methods for measuring adherence are lacking, with current studies relying on heterogeneous approaches such as self-reporting, electronic monitoring, or pharmacy refill data, which limits comparability across clinical trials and real-world cohorts [5].
2. Additional real-world evidence is needed to evaluate adherence and patients satisfaction before and after switching from daily rhGH to LAGH, as most available data come from short-term studies or open-label extensions phases. In this context, international registries such as GloBE-Reg, which include adherence in their agreed minimum dataset for monitoring rhGH therapy in GHD, will be crucial to generate long-term comparative data on adherence and patient-reported burden across daily and long-acting GH, even though adherence is recognised as important but inherently difficult to capture consistently in routine practice [27, 72].
3. The long-term impact of LAGH on final adult height and metabolic outcomes remains uncertain, as most trials have focused on short- to medium-term growth velocity and IGF-1 responses [23]. In pediatric studies, BMI SDS generally remains within the normal range; however, in a recent 4-year analysis of once-weekly somapacitan, mean BMI SDS increased significantly over time, although values remained within normal limits at the group level [73]. However, no published pediatric LAGH study has reported Dual Energy X-ray Absorptiometry (DXA)-derived or other detailed body-composition outcomes, leaving long-term effects on lean and fat mass unknown. Adult LAGH studies with somapacitan and lonapegsomatropin have demonstrated DXA-confirmed improvements in body composition [65, 74], but DXA is seldom performed in children because of radiation exposure and feasibility constraints; alternatives such as Bioelectrical Impedance Analysis (BIA), ultrasound, or MRI may be more appropriate for future pediatric evaluations.
4. Issues of access and equity are likely to influence broader LAGH adoption. Economic considerations, reimbursement policies, and healthcare infrastructure might create disparities across regions and patient groups [23]. In this context, real-world economic evaluations provide important insights, and recent US budget impact analysis estimated that introducing lonapegsomatropin could reduce overall costs by 959,629 dollars over five years—equivalent to savings of 3,283 dollars per patient per month—mainly due to reduced acquisition costs and lower product waste compared with daily therapy [75]. However, these estimates rely on assumptions about GH wastage at the end of each pen or cartridge that may not fully reflect real-world practice, particularly in US settings where pharmacy benefit managers and insurers often restrict overfilling and wastage. As a result, the projected cost savings in this model are likely to represent a theoretical upper bound rather than the actual budget impact in many healthcare systems. Therefore, current pharmacoeconomic data should be viewed cautiously and confirmed by independent real-world cost analyses using empiric wastage and utilization patterns.
5. Most of the available evidence derives from randomized clinical trials and observational studies sponsored by pharmaceutical companies, which may introduce subtle biases in study design, conduct, reporting, and publication. Although conflicts of interest and funding sources are clearly stated, this sponsorship should be kept in mind when interpreting the findings of the present review.
6. No study to date has systematically examined the clinical and biochemical consequences of poor adherence specifically to LAGH regimens. Analyses linking dose histories to auxological and IGF-1 outcomes have been conducted almost exclusively in the context of daily rhGH, and it remains unknown whether the same adherence thresholds and patterns apply to weekly preparations.
7. Shared decision-making remains essential to optimize GH therapy. Physicians should support families in navigating therapeutic options by integrating efficacy and safety data with lifestyle factors, preferences, and psychosocial context [5]. Personalized guidance and open communication will be key to realizing the full potential of LAGH therapy in clinical practice.

## Conclusions

Adherence remains a cornerstone of all successful treatments, including GH therapy. While daily rhGH is highly effective, its demanding injection schedule often leads to suboptimal adherence in real-world settings. LAGH formulations demonstrably reduce injection frequency and perceived treatment burden, and preliminary data suggest potential advantages in satisfaction and persistence compared with daily rhGH. However, current evidence on adherence is indirect, heterogeneous, and largely derived from controlled trial settings, so no firm conclusions can yet be drawn regarding the magnitude of any adherence benefit or its impact on auxological and biochemical outcomes: LAGH was introduced with the explicit aim of improving adherence, yet this outcome has been only marginally investigated.

Transitioning from daily to weekly GH therapy should therefore be viewed as a promising strategy to be rigorously tested, rather than a proven solution, and future research should prioritize longitudinal real-world studies with standardized adherence metrics. In the meantime, clinicians may reasonably consider LAGH within a broader adherence-focused strategy that includes careful patient selection, shared decision-making, and ongoing monitoring, while being aware of the important knowledge gaps that remain.

## Data Availability

No datasets were generated or analysed during the current study.
